# A systematic review of deep brain stimulation for substance use disorders

**DOI:** 10.1038/s41398-024-03060-1

**Published:** 2024-09-06

**Authors:** David Zammit Dimech, Audrey-Ann Zammit Dimech, Mark Hughes, Ludvic Zrinzo

**Affiliations:** 1 University of Edinburgh, Clinical & Surgical Sciences, Edinburgh, UK; 2 Centre for Clinical Brain Sciences, University of Edinburgh, Edinburgh, UK; 3 UCL Institute of Neurology, Functional Neurosurgery Unit, Department of Clinical & Motor Neurosciences, University College London, London, UK

**Keywords:** Human behaviour, Scientific community

## Abstract

**Background:**

Pharmaco-psychiatric techniques remain the mainstay, first line treatments in substance use disorders (SUD), assisting in detoxification but largely ineffective at reducing dependence. The path to rehabilitation and freedom from addiction often proves uncertain and laborious for both patients and their significant others. Relapse rates for multiple substances of abuse are considerable and the number of SUD patients is on the increase worldwide.

**Objective:**

To assess efficacy of deep brain stimulation (DBS) as a therapeutic solution for SUDs.

**Methods:**

A systematic electronic database search of PubMed and EMBASE retrieved DBS addiction-focused studies on humans, of which a total of 26 (*n* = 71) from 2007 to 2023 were deemed eligible, including the first randomized controlled trial (RCT) in this field. This review was prospectively registered with PROSPERO: CRD42023411631.

**Results:**

In addressing SUDs, DBS targeting primarily the nucleus accumbens (NAcc), with or without the anterior limb of the internal capsule, presented encouraging levels of efficacy in reducing cravings and consumption, followed by remission in some subjects, but still reporting relapses in 73.2% of patients.

**Conclusions:**

For treatment-refractory addictions DBS use seems limited to reducing cravings with a satisfactory degree of success, yet not clinically consistent in inducing abstinence, suggesting involvement of factors unaffected by DBS intervention. Furthermore, costs and the scale of the problem are such that DBS is unlikely to have a significant societal impact. Nevertheless, DBS may provide insight into the biology of addiction and is worthy of further research using increased methodological rigor, standardized outcome measures, and pre-established surgical protocols.

## Introduction

Substance addiction is a chronic relapsing disorder, with compulsive behaviors for seeking and consuming substances that cause dependence, persisting regardless of negative consequences [1]. It encompasses an element of impulsivity, and is defined as “a failure to resist an impulse, drive or temptation to perform an act that is harmful to the person or others” [2]. Addiction is a functional brain pathology demonstrating behavioral anomalies when interacting with the substance of abuse, characterized by progressively less controllable compulsions, resulting in deleterious physiological and psychosocial consequences. Frequent, careless and irresponsible use of the substance causes refocusing of priorities that privilege the addiction over duties and other activities [3].

## The social burden of addiction

On a global scale, substance use disorders (SUDs) are a leading contributor to morbidity and mortality, generating severe health concerns and significant hardship for those afflicted by the addiction and their significant others [4]. A 2020 US national survey found that 38.7 million individuals suffered from a SUD [5]. Substances commonly subject to abuse and the creation of dependence include alcohol, opioids, cocaine, nicotine, amphetamines, methamphetamines, cannabis, hallucinogens, sedatives and tranquilizers [2]. In the West, 25% of deaths are connected to use of psychotropic substances. Worldwide, 284 million individuals aged between 15 and 64 abused drugs in 2020 [6, 7]. 21st century US victims of drug overdose are over 1 million. In 2021, 88% of them were ascribed to synthetic opioids, namely fentanyl [8]. In the EU, heroin is the major cause of drug-induced fatalities [9, 10] but a shift towards fentanyl, the main cause of the US opioid epidemic, is noted [11, 12].

Alcohol and nicotine are the substances of abuse responsible for the highest number of deaths worldwide. The heaviest consumption of alcohol occurs in Europe, with 10.1% of all deaths and 10.8% of all DALY attributable to alcohol [13, 14]. The financial burden on 48 countries, including the G20, EU and OECD countries, was projected at a loss of $1.6 trillion yearly from 2020 to 2050 [15]. In the past century nicotine claimed the lives of about 100 million individuals [16]. There are 1.13 billion smokers worldwide with half expected to die due to nicotine addiction, as each lifetime smoker loses at least 10 years of life [17]. 3000 daily deaths occur in China, both the biggest manufacturer and the largest consumer of tobacco [18]. Nicotine use is responsible for enough years lived with disability to account for over 25% of productive years lost in individuals aged between 45 and 79. The lost productivity and accompanying health care costs derived from morbidity and mortality amounts to $2 trillion every year [17].

The burden of substance addiction on society is substantial. Despite efforts at establishing treatment practices that adequately address SUDs, reported efficacy has been disappointing. Relapse rates fall within the same lamentable range, between 50% and 70% [19]. This has prompted the need for the exploration of new therapeutic venues. The clinical application of neuromodulation has yielded satisfactory results for psychiatric and movement disorders, and may hold promise for the treatment of addiction [20].

## Neurosurgical interventions to treat addiction

Before 1960, pioneering experiments to treat addiction disorders were largely imprecise, with intentional brain lesions targeting ambiguous or widespread areas that resulted in unwanted, irreversible difficulties with cognition, speech and other functions [21]. Leucotomies, and later hypothalamotomies, were performed in Germany, Sweden and Russia [22]. In 1978, stereotactic cingulotomies in Indian patients with an alcohol addiction reported a 68% abstinence rate [23]. Before being banned, cingulotomies in Russia between 1999 and 2002, reported a 30% sudden remission in heroin users [24, 25]. Similarly, in China from 2000 to November 2004, when the practice was outlawed, bilateral NAcc ablation had resulted in a reduction in relapse rates of 57.5% in opioid addicts 15 months after surgery, [26] while another Chinese study reported a 5-year abstinence of 47.4% in 60 patients [27, 28]. Neural ablation procedures were followed by enhanced DBS techniques that target specific areas, with the NAcc garnering major interest. Located in the basal forebrain and positioned between the caudate and the putamen, the NAcc is a substructure, and the main component, of the ventral striatum within the basal ganglia [29].

## A role for deep brain stimulation

Deep brain stimulation (DBS) is an invasive neurosurgical intervention via which there is stereotactic implantation of either unilateral or bilateral electrodes targeting defined brain nuclei involved in specific neural activity [30]. They will regulate abnormal impulses or elicit neurotransmitter release depending on the area stimulated [31], triggering neuroanatomical remodeling on a cellular level [32] and thus generating an effect in terms of neuroplasticity [33, 34]. The implanted electrodes are connected via tunneled wires passing subcutaneously to a neurostimulator located in the subclavicular region underneath the pectoral muscles [35, 36]. The pulse generator will deliver electrical stimulation and thereby provide direct communication with neurons and cortico-striatal circuits [37, 38]. The pulse generator can be programmed depending on the planned treatment, the specific area being targeted, feedback from the patient and the desired therapeutic response [39].

The Food and Drug Administration (FDA) has, to date, approved DBS for the treatment of essential tremor and severe tremor in Parkinson’s Disease in 1997, and of motor symptoms in advanced Parkinson’s Disease in 2002, dystonia in 2003, obsessive-compulsive disorder (OCD) in 2009 and epilepsy in 2018 [20]. Although not FDA-approved, DBS is also used in treating Tourette syndrome, chronic pain, major depressive disorder, anorexia nervosa, obesity, migraine and cluster headaches [40, 41]. The possibility of targeting the nucleus basalis of Meynert to treat Azlheimer’s Disease is also being explored [42]. Use of DBS to treat SUDs is a relatively novel approach deserving of assessment, given results with pharmaco-psychiatric therapy.

## Methods

### Search strategy

The systematic review was prospectively registered with PROSPERO (ID: CRD42023411631) and reported according to Preferred Reporting Items for Systematic Reviews and Meta-Analyses (PRISMA) 2020 guidelines [43]. The databases consulted were PubMed and Embase, up until August 2023. Advanced search strategies were employed in mining for data and included Medical Subject Headings (MeSH) terms in PubMed: “Deep Brain Stimulation”, “Behavior, Addictive”, “Substance-Related Disorders”, “Opioid-Related Disorders”, “Cocaine-Related Disorders”, “Amphetamine-Related Disorders”, “Tobacco Use Disorder”, “Morphine Dependence”, “Heroin Dependence”, “Opium Dependence”, “Alcoholism” and “Narcotic-Related Disorders”, and a comprehensive list of terms in Embase which encompassed ‘deep brain stimulation/’, ‘addiction medicine/’, ‘substance-related disorders/’, ‘alcohol-related disorders/’, ‘amphetamine-related disorders/’, ‘cocaine-related disorders/’, ‘drug overdose/’, ‘inhalant abuse/’, ‘substance abuse, intravenous/’, ‘substance abuse, oral/’, ‘substance withdrawal syndrome/’, ‘tobacco use disorder/’ and ‘opioid-related disorders/’.

### Selection criteria and process

Records retrieved were assessed in accordance with predefined inclusion and exclusion criteria. All DBS trials conducted on human beings for addiction disorders were included. The substance of concern could be any that may lead to dependence. DBS studies which were focused on other neuropsychiatric disorders, including eating disorders and mood disorders, or those focused on neurodegenerative disorders, were excluded. Studies not concerning DBS as an intervention for an addiction disorder were excluded. Only publications reported in English were considered. Animal studies were excluded. Database search results were exported to a spreadsheet, removing duplicates. Detection tools were utilized to exclude studies in Excel. Two researchers independently screened publication titles and later abstracts and full texts of the filtered studies.

### Data collection

When available in the publications selected, the following data items were noted: first author, year of publication, study location, study design, sample size, patient age and sex, substance being treated for, DBS target area, DBS technical parameters including frequency, pulse width and amplitude, laterality, comorbid psychiatric pathologies, outcomes including treatment response, quality of life (QoL) parameters and adverse events, and length of follow-up period. Data was collected by two reviewers, working in conjunction.

### Quality assessment

For the single RCT identified, the Cochrane risk-of-bias (RoB2) tool was used, identifying a low risk of bias on all five domains evaluated [44]. The evaluation for quality and risk of bias for the non-randomized studies retrieved was performed using the Methodological Index for Non-Randomized Studies (MINORS) [45]. Two researchers independently reviewed the publications, assessing their quality and categorizing them. All eligible studies identified were deemed adequate. They were read in their entirety before being included in the review.

### Outcome evaluation

Abstinence and relapse episodes are a readily accessible, but incomplete, measure of success of DBS interventions within the context of overcoming SUDs. Benefits intimately associated with the patients’ recovery from dependence, including improved neuropsychiatric states, reduced cravings and consumption, were also reported in concomitance with non-abstinent states. Abstinence was therefore not considered the primary outcome measurement. Instead, for the purpose of providing a more comprehensive outlook, results analysis encompassed the aforementioned benefits, and data is reported for abstinence, a full relapse, and for a partial relapse in which the patient would not have attained abstinence yet still reported improvements when compared to pre-operative conditions.

## Results

### Study selection and characteristics

**Fig. 1:**
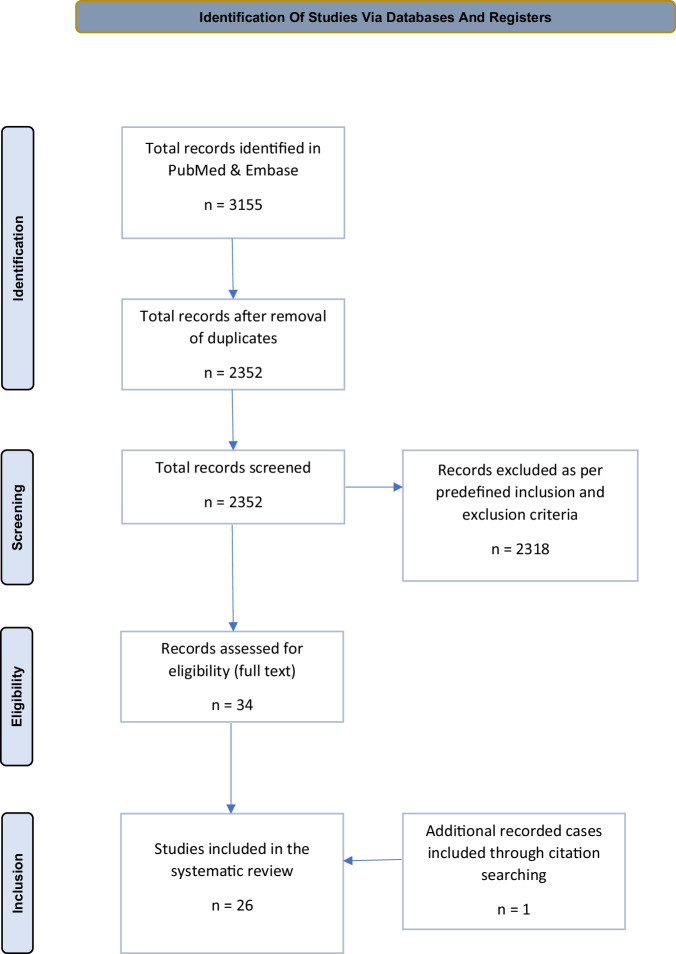
Preferred Reporting Items for Systematic Reviews and Meta-Analyses (PRISMA) flowchart according to 2020 guidelines, showing the phases of study [43].

The records deemed eligible were 25, spanning from 2007 to 2023. An additional case was included following citation searching. There was one double-blind RCT [23] and 25 non-RCTs. The PRISMA flowchart is visible in Fig. 1. All publications retrieved were small scale studies, reporting results for one or a small group of patients. The total number of individual patients treated was 71, with some reported in multiple studies. Patient overlap, alongside other relevant data, is indicated in Table 1 [46–71]. Twelve patients were female. Ages ranged from 22 to 69.

**Table 1:** Summary of characteristics of DBS addiction studies in humans, presented in a chronological order.

Authors	Study	Patients	Age	Sex	Addiction	Target	Parameters ^+^	Laterality	Follow-up
Kuhn et al. [53]	Case report	1	54	M	Alcohol	NAcc	130 Hz; 90 μs; 3–4.5 V	Bilateral	1 year
Xu et al. [54]	Case report	1	24	M	Heroin	NAcc	145 Hz; 90 μs; 2.5 V	Bilateral	2 years
Muller et al. [55]	Case series	3	36-40	M	Alcohol	NAcc	130 Hz; 90 μs; 3.5–4.5 V	Bilateral	1 year
Kuhn et al. [49]	Case series	10	28-58	F: 3 M: 7	Nicotine	NAcc	130–145 Hz; 90, 180 μs; 3–6.5 V	Unilateral: 5 Bilateral: 5	30 months
Mantione et al. [50]	Case report	1	47	F	Nicotine	NAcc	185 Hz; 90 μs; 3.5 V	Bilateral	2 years
Zhou et al. [56]	Case report	1	24	M	Heroin	NAcc	145 Hz; - μs; 0.8–2.5 V	Bilateral	6 years
Kuhn et al. [57]	Case report	1	69	M	Alcohol	NAcc	130 Hz; 120 μs; 5.5 V	Bilateral	1 year
Valencia-Alfonso et al. [58]	Case report	1	47	M	Heroin	NAcc	180 Hz; 90 μs; 3.5 V	Bilateral	10 months
Heldman et al. [59]	Case report	1^a^	38	M	Alcohol	NAcc	130 Hz; 90 μs; 3.5 V	Bilateral	2 years
Voges et al. [60]	Case series	5^b^	36-65	M	Alcohol	NAcc	130 Hz; 90 μs; 3.5–4.5 V	Bilateral	x̄ 3.1 years (max: 4 yrs)
Kuhn et al. [61]	Case report	2	31, 33	F, M	Heroin	NAcc	130–140 Hz; 90–120 μs; 4.5–5 V	Bilateral	2 years
Gonçalves-Ferreira et al. [47]	N-of-1 trial	1	36	M	Cocaine	NAcc, BNST, ALIC	Right: 150 Hz; 150 μs; 3–4 V Left: 150 Hz; 150 μs; 2.5–3 V	Bilateral	30 months
Muller et al. [62]	Case series	5^c^	35-55	M	Alcohol	NAcc	130 Hz; 90 μs; 3.5–4.5 V	Bilateral	8 years
Ge et al. [63]	Open-label pilot	7	26-50	F: 1 M: 6	Heroin	NAcc, ALIC	145–185 Hz; 150–240 μs; 2–3.3 V	Bilateral	2 years (max: 40 months)
Zhang et al. [64]	Case report	1	39	M	Heroin	VC, VS	130 Hz; 90 μs; 2.5–5.5 V	Bilateral	105 days
Zhang et al. [51]	Case report	1	33	M	Methamphetamines	NAcc, VC	130 Hz; 210 μs; 3 V	Bilateral	1 year
Chen et al. [65]	Open-label pilot	8^d^	22-50	F: 1 M: 7	Heroin	NAcc, ALIC	130–185 Hz; 150–240 μs; 1.5–7 V	Bilateral	2 years
Ge et al. [52]	Case report	2	38, 49	M	Methamphetamines	NAcc, ALIC	150, 165 Hz; 210, 240 μs; 2.5,3.3 V	Bilateral	30 months
Zhang et al. [66]	Case report	1	42	M	Heroin	NAcc	-	Bilateral	1 year
Zhu et al. [67]	Case report	1	28	M	Buccinazine, Morphine, Hypnotics	NAcc, AC	145–160 Hz; 90 μs; 2.7–2.8 V	Bilateral	1 year
Leong et al. [68]	Open-label pilot	8	32-63	F: 4 M: 4	Alcohol	ACC	-	Bilateral	48 weeks
Mahoney et al. [69]	Case report	1	30	M	Opioids, Benzodiazepines	NAcc, VC	145 Hz; 90 μs; 6 V	Bilateral	12 weeks + 1 year
Davidson et al. [70]	Open-label pilot	6	30-66	F: 2 M: 4	Alcohol	NAcc	130 Hz; 90 μs; 3.5–4.5 V	Bilateral	1 year
Bach et al. [46]	RCT	12	DBS: x̄ 44.2, Control: x̄ 47.7	M	Alcohol	NAcc, ALIC	130 Hz; 90 μs; 3.5–4.5 V	Bilateral	Blind: 6mo N/Blind: 12 m
Rezai et al. [71]	Open-label pilot	4^e^	22-44	M	Opioids	NAcc,VC	125–145 Hz; 90–300 μ; 3.0–4.5 V	Bilateral	12 weeks + 1 year
Vorspan et al. [48]	Case report	1	40 s	M	Cocaine	StN	130 Hz; 60 μs; 1.25–3 V	Bilateral	2 years

*StN* subthalamic nucleus, *NAcc* nucleus accumbens, *BNST* bed nucleus of the stria terminalis, *ALIC* anterior limb of internal capsule, *ACC* anterior cingulate cortex, *VC* ventral capsule, *VS* ventral striatum, *AC* anterior capsulotomy, *Hz* hertz, *μsec* microsecond, *V* Volts, *RCT* randomized controlled trial, *x̄* Mean, *yrs* years.

^+^Parameters include frequency, pulse width and amplitude presented in that order.

^a^patient reported earlier in Muller et al. [55].

^b^3 of 5 patients reported earlier in Muller et al. [55].

^c^3 of 5 patients reported earlier in Muller et al. [55]; 2 of 5 patients reported earlier in Voges et al. [60]

^d^5 of 8 patients reported earlier in Ge et al. [63].

^e^1 of 4 patients reported earlier in Mahoney et al. [69].

Patients voluntarily subjecting themselves for surgery had several years of substance abuse behind them, with a history of unsuccessful pharmacotherapeutic interventions. Eleven publications addressed addiction for opioids while nine focused on alcohol use disorders. Two studies focused on cocaine addiction [47, 48], two on nicotine [49, 50], and two involved abuse of methamphetamines [51, 52].

### Risk of bias

RCT assessment using RoB2 demonstrated a low risk of bias with a detailed randomization process, a strict protocol for interventions, a six-month blinding period, complete data for primary outcomes of all participants and for secondary outcomes for most participants, validated measures and blinded assessments. Despite the challenging recruitment that resulted in a small sample size, the methodology was robust and reporting transparent. The observational studies were assessed via MINORS, resulting in adequate scores. A few MINORS categories were not applicable to most studies given the study design of most publications but overall, they scored well on stating objectives, endpoints and their assessment, as well as follow-up.

### Target area and stimulation parameters

The target area in the brain varied slightly, focusing mostly on targets around the ventral capsule, including the NAcc and the bed nucleus of the stria terminalis (BNST). In most cases, researchers described intervention on the NAcc, with exceptions including the BNST [47], anterior cingulate cortex [68], and subthalamic nucleus [48]. In all cases except one [26], lead implantation was performed bilaterally. The electrical stimulation parameters applied to patients varied in frequency, pulse width and amplitude. In some studies, the starting parameters were modified in attempts to adjust to the individual patients’ perceived therapeutic needs, such as insomnia [65]. In one instance anxiety and hypomania at 4.5 Volts were controlled at 3.7 Volts, with a further reduction to 3.3 Volts addressing insomnia and teeth grinding [52]. Kuhn et al. [49] had reported increased efficacy of DBS in tobacco use disorder patients on application of higher voltage [49]. In subsequent studies by Kuhn et al. [57, 61], however, the maximum voltage was lowered for alcohol and heroin dependence [57, 61]. The highest amplitude setting was noted in Chen et al. [65], 7 Volts, but this did not prevent the two patients receiving high voltage stimulation from eventually relapsing [65]. Pulse stimulation frequency across studies ranged from 130 to 185 Hertz. Pulse width ranged between 90 and 240 μsec. Data described in this section is present in Table 1.

### Benefits of DBS

Although at varying degrees, studies reported therapeutic benefits for most of the patients. Even in Zhang et al. [64], where the patient passed away about 100 days from surgery due to a heroin overdose, cravings were reduced [64]. Whether this could be attributed to acquiescence bias by the patient cannot be determined, especially given the tragic conclusion of the trial. Although most patients (73.2%) did not achieve complete abstinence, many reported reductions in cravings and in episodes of heavy consumption. Whether these improvements translated into practical long-term psychosocial benefits for the patients was not reported in most publications, although some mention patients finding employment [52, 55, 60, 69, 71], marrying and having children [52] or improving family ties [71]. Others report the deaths of patients, unrelated to surgery [62, 64, 70]. A summary of patient outcomes is available in Table 2.

**Table 2:** Summary of clinical outcomes of DBS addiction studies in humans, presented according to substance.

Authors	Patients	Follow-up	Outcomes
**Alcohol**			
Kuhn et al. [53]	1	1 year	Greatly reduced cravings and alcohol use.
Muller et al. [55]	3	1 year	Immediate reduction of cravings in all patients. 2 of 3 patients abstinent. 1 patient abstinent for most of the follow-up period but relapsed, with reduced consumption despite being in jail.
Kuhn et al. [57]	1	1 year	By the 8th month there was a significant reduction in alcohol use. The patient was abstinent at the 1-year follow-up check point.
Heldman et al. [59]	1	2 years	The patient remained abstinent. Reduced risk-taking behavior.
Voges et al. [60]	5	x̄ 3.1 years (max: 4 yrs)	2 patients remained abstinent. 2 patients relapsed infrequently and reduced overall consumption. 1 patient abstinent until 16 months, with reduced cravings, then relapsed due to bilateral electrode dislocation.
Muller et al. [62]	5	8 years	1 patient abstinent for 8 years. 1 patient abstinent for 6 years then lost to follow-up. 1 patient reduced consumption, relapsed, died due to other causes after 4 years. 1 patient relapsed after 16 months, electrode dislocation after 30 months, died due to other cause after 8 years. 1 patient reduced consumption, relapsed.
Leong et al. [68]	8	48 weeks	All patients abstinent at least 6 months. 2 participants relapsed at the 48 week follow-up. Alcohol cravings reduced by 60.7%. Alcohol consumption reduced by 80%. Depression reduced by 63.5%. No changes in anxiety.
Davidson et al. [70]	6	1 year	Patients experienced reduced cravings. 5 of 6 patients greatly reduced consumption. 1 of these 5 patients stated she drank due to habit but had reduced consumption by a third. Improved mood disorders, especially anxiety, except for 1 patient who remained severely depressed throughout. This was the only patient to not reduce consumption despite reduced cravings. After being abstinent for 6 months he relapsed and also required detoxification. 1 patient died 14 months post-op from a myocardial infarction.
Bach et al. [46]	12	Blinded: 6 months Unblinded: 12 months	For first 6 months DBS vs control. For additional 12 months all DBS on. Increased abstinent days (first alcohol use mean time during first 6 months: DBS = 70.5 days, control = 29.7 days; but no significant difference) reduced cravings, reduced heavy drinking days, improved mood in DBS patients compared to controls. 1 DBS patient remained abstinent for 6 months, and 1 for 5 months. During additional 12 months with all patients on DBS there was no difference in mean time to first alcohol use.
**Opioids**			
Xu et al. [54]	1	2 years	The patient remained abstinent.
Zhou et al. [56]	1	6 years	The patient remained abstinent for the full 6 year follow-up. He regained a healthy weight and showed improvement in cognitive functions. Electrode removal after 3 years from operation.
Valencia-Alfonso et al. [58]	1	10 months	Reduced consumption initially, then abstinence for 6 months except for a 2 week period.
Kuhn et al. [61]	2	2 years	The patients remained abstinent, except for a single episode a few weeks after surgery. Reported reduced cravings. They consumed amphetamines for weight management or out of boredom, but in general presented improved mood disorders. Worsening was due to 1 patient requiring a battery change.
Ge et al. [63]	7	2 years (max: 40 months)	4 patients abstinent after 40, 35, 23 and 21 months respectively. 2 patients relapsed after 7 and 10 months of abstinence. 1 patient lost to follow-up at 3 months. Improved mood and vigor in abstinent patients.
Zhang et al. [64]	1	105 days	Early improvement. Gradual drug cravings and consequent relapses 2 months after. Death from heroin overdose.
Chen et al. [65]	8	2 years	5 patients abstinent after 3 years. 2 patients relapsed after 7 and 10 months of abstinence. 1 patient lost to follow-up at 3 months. In abstinent patients: improved psychiatric disorders, sexual life, QoL. Gained weight. 1 got married. 2 conceived children. No positive effects, changes noted in relapsed patients.
Zhang et al. [66]	1	1 year	Remained abstinent except for a relapse episode halfway through the follow-up period at 6 months.
Zhu et al. [67]	1	1 year	Cravings reduced by 3 months. Patient abstinent by 1 year. Improvement in mood disorders, cognition, insomnia.
Mahoney et al. [69]	1	12 weeks + 1 year	Patient remained abstinent. Improved cravings, executive function and mood disorders.
Rezai et al. [71]	4	12 weeks + 1 year	2 patients abstinent for >1150 and >520 days. 1 participant had reduced cravings and anxiety but was not compliant and dropped out early. 1 patient relapsed but with reduced frequency and less adverse consequences.
**Nicotine**			
Kuhn et al. [49]	10	30 months	3 patients remained abstinent. The rest relapsed.
Mantione et al. [50]	1	2 years	The patient relapsed until the 10th month post-op, when OCD was eventually managed. The patient was then abstinent and showed improvement in weight management, OCD and mood disorders.
**Cocaine**			
Goncalves-Ferreira et al. (2016)	1	30 months	Significant reduction in dependence and use of cocaine.
Vorspan et al. [48]	1	2 years	3 double-blind crossovers did not demonstrate correlation with abstinence periods. No craving reduction.
**Methamphetamines**			
Zhang et al. [51]	1	1 year	Abstinent for 1 year. Reduced cravings. Improved mood disorder.
Ge et al. [52]	2	30 months	1 patient remained abstinent, gained weight and improved his sexual life. 1 patient relapsed but showed displaced electrode on CT and MRI, not implanted accurately in nucleus accumbens.

### Relapses post-DBS

Some patients did go on to present a largely unchanged situation or a consumption of substances other than the one being treated for. Apart from the aforementioned fatal overdose [64], Voges et al. [60] and Muller et al. [62] report on the same individual having stayed abstinent from alcohol for 16 months until a series of relapses [60, 62]. He coincidentally re-presented following a generalized seizure after being lost to follow-up, where it was radiographically confirmed that electrode dislocation had occurred. Three patients reported in both Ge et al. [63] and Chen et al. [65] also demonstrated only temporary improvement [63, 65]. One of the patients being treated for methamphetamine use in Ge et al. [52] relapsed at 6 months but CT and MRI scans had confirmed that one of the electrodes was not accurately implanted in the NAcc [52]. One of the six patients enrolled by Davidson et al. [70] consistently reported reduced alcohol cravings and was abstinent for 6 months, but severe depression persisted with multiple relapses and the need for detoxification [70]. In Kuhn et al. [61], although patients being treated for heroin addiction remained abstinent for the opioid and presented improved mood, they both reported amphetamine consumption [61]. One of the patients engaged in significant alcohol use after almost 2 years post-op. However, it was later reported that he required a battery change. Kuhn et al. [49] reported seven full relapses [49], Leong et al. [68] two within 1 year [68] and Rezai et al. [71] one [71].

### Success rates

Table 3 provides data about abstinence, partial relapses and full relapses. Patients considered to have relapsed completely totaled 23.9% (*n* = 17), seven of whom were from one study exploring nicotine addiction [26]. 26.8% of patients (*n* = 19) remained abstinent throughout follow-up while 49.3% (*n* = 35) exhibited occasional relapses. Table 4 presents this data for five distinct subgroups categorized by substance of abuse, providing a more immediate and clear picture of results achieved. It is important to note that the follow-up period varied quite considerably between the studies, ranging from around 100 days to 8 years. This impacts on the end-state report for the patients, with longer follow-up periods expressing increased scientific value.

**Table 3:** Numerical representation of patients and outcomes for DBS addiction studies in humans.

Authors	Follow-up	Patients	Non-overlapping patients	Abstinent	Partial relapse	Full relapse
Kuhn et al. [53]	1 year	1	1		1	
Xu et al. [54]	2 years	1	1	1		
Muller et al. [55]	1 year	3	3	2	1	
Kuhn et al. [49]	30 months	10	10	3		7
Mantione et al. [50]	2 years	1	1		1	
Zhou et al. [56]	6 years	1	1	1		
Kuhn et al. [57]	1 year	1	1		1	
Valencia-Alfonso et al. [58]	10 months	1	1		1	
Heldman et al. [59]	2 years	1	0			
Voges et al. [60]	x̄ 3.1 years (max: 4 yrs)	5	2		1	1
Kuhn et al. [61]	2 years	2	2		2	
Goncalves-Ferreira et al. (2016)	30 months	1	1		1	
Muller et al. [62]	8 years	5	0			
Ge et al. [63]	2 years (max: 40 months)	7	7	4		3
Zhang et al. [64]	105 days	1	1			1
Zhang et al. [51]	1 year	1	1	1		
Chen et al. [65]	2 years	8	3	3		
Ge et al. [52]	30 months	2	2	1		1
Zhang et al. [66]	1 year	1	1		1	
Zhu et al. [67]	1 year	1	1		1	
Leong et al. [68]	48 weeks	8	8		6	2
Mahoney et al. [69]	12 wks + 1 yr	1	1	1		
Davidson et al. [70]	1 year	6	6		5	1
Bach et al. [46]	6 + 12 months	12	12	1	11	
Rezai et al. [71]	12 wks + 1 yr	4	3	1	1	1
Vorspan et al. [48]	2 years	1	1		1	
**Patients (numbers):**			**71**	**19**	**35**	**17**
**Patients (percentages):**			**100%**	**26.80%**	**49.30%**	**23.90%**

**Table 4:** Numerical representation of patients and outcomes for DBS addiction studies according to substance.

Authors	Follow-up	Patients	Non-overlapping patients	Abstinent	Partial relapse	Full relapse
**Alcohol**						
Kuhn et al. [53]	1 year	1	1		1	
Muller et al. [55]	1 year	3	3	2	1	
Kuhn et al. [57]	1 year	1	1		1	
Heldman et al. [59]	2 years	1	0			
Voges et al. [60]	x̄ 3.1 years (max: 4 yrs)	5	2		1	1
Muller et al. [62]	8 years	5	0			
Leong et al. [68]	48 weeks	8	8		6	2
Davidson et al. [70]	1 year	6	6		5	1
Bach et al. [46]	6 + 12 months	12	12	1	11	
**Patients (numbers) [alcohol]:**			**33**	**3**	**26**	**4**
**Patients (percentages) [alcohol]:**			**100%**	**9.10%**	**78.80%**	**12.10%**
**Opioids**						
Xu et al. [54]	2 years	1	1	1		
Zhou et al. [56]	6 years	1	1	1		
Valencia-Alfonso et al. [58]	10 months	1	1		1	
Kuhn et al. [61]	2 years	2	2		2	
Ge et al. [63]	2 years (max: 40 months)	7	7	4		3
Zhang et al. [64]	105 days	1	1			1
Chen et al. [65]	2 years	8	3	3		
Zhang et al. [66]	1 year	1	1		1	
Zhu et al. [67]	1 year	1	1		1	
Mahoney et al. [69]	12 wks + 1 yr	1	1	1		
Rezai et al. [71]	12 wks + 1 yr	4	3	1	1	1
**Patients (numbers) [opioids]:**			**22**	**11**	**6**	**5**
**Patients (percentages) [opioids]:**			**100%**	**50%**	**27.30%**	**22.70%**
**Nicotine**						
Kuhn et al. [49]	30 months	10	10	3		7
Mantione et al. [50]	2 years	1	1		1	
**Patients (numbers) [nicotine]:**			**11**	**3**	**1**	**7**
**Patients (percentages) [nicotine]:**			**100%**	**27.30%**	**9.10%**	**63.60%**
**Cocaine**						
Goncalves-Ferreira et al. (2016)	30 months	1	1		1	
Vorspan et al. [48]	2 years	1	1		1	
**Patients (numbers) [cocaine]:**			**2**	**0**	**2**	**0**
**Patients (percentages) [cocaine]:**			**100%**	**0%**	**100%**	**0%**
**Methamphetamines**						
Zhang et al. [51]	1 year	1	1	1		
Ge et al. [52]	30 months	2	2	1		1
**Patients (numbers) [meth]:**			**3**	**2**	**0**	**1**
**Patients (percentages) [meth]:**			**100%**	**66.70%**	**0%**	**33.30%**

### Neuropsychiatric benefits

Comorbid psychiatric diagnoses were reported in most publications (*n* = 21) [46, 47, 49–53, 55–57, 60–65, 67–71], with many indicating post-op improvement. Some patients, having been embroiled in a long-term addiction pathology, and having failed multiple attempts at remission via more conservative means, carried the burden of numerous concurrent neuropsychiatric disorders including depression, anxiety, agoraphobia, eating disorders, Tourette’s syndrome, OCD, antisocial personality disorder, panic disorder, post-traumatic stress disorder, bipolar disorder, anhedonia and sleeping disorders.

The various assessment tools used to gauge presence and severity of neuropsychiatric disorders included the Beck Depression Inventory (BDI) [46, 53, 61, 67, 70], State-Trait Anxiety Inventory [46, 53, 68], Minnesota Multiphasic Personality Inventory [54, 56], Symptom Checklist 90 [52, 55, 56, 59, 60, 62, 65], Yale-Brown Obsessive Compulsive Scale [47, 50, 63, 65], Hamilton Anxiety Scale [46, 50, 61, 67], Hamilton Depression Scale (HAMD) [46, 50, 52, 63, 65, 67, 70], Self-Rating Depression Scale [56], Self-Rating Anxiety Scale [59], Montgomery-Åsberg Depression Rating Scale [47, 48], Clinical Global Impressions Scale [47], Global Severity Index [60], Young Mania Rating Scale [67], Beck Anxiety Inventory [67, 70], Pittsburgh Sleep Quality Index [67], Global Assessment of Functioning Scale (GAF) [46], Chapman Anhedonia Scale [46], and the Snaith-Hamilton Pleasure Scale (SHAPS) [46].

From the eight patients in Chen et al. [65] three were diagnosed with OCD, one with depression and two presented with both [65]. Of the latter, both relapsed after 6 months and showed no improvement in neuropsychiatric symptoms or QoL. Benefits were reported for five patients, who all gained weight (except for one female), had improved sexual lives, neuropsychiatric symptoms, and QoL. In Kuhn et al. [61] there was marked improvement in mood disorder for one patient but progressive worsening in the other, accompanied by use of alcohol and amphetamines [61]. The issue improved after replacement of the impulse generator. One patient in Ge et al. [52] relapsed, with accompanying depressive symptoms, although the authors attributed it to inaccurate lead placement [52]. Bach et al. [46] reported reduced anhedonia and depression, and improved QoL for patients whose implanted neurostimulator was on early in the trial, but no improvement for those on sham stimulation [46]. After 6 months, when the DBS vs control phase was over, the SHAPS mean scores were 0.8 vs 6.2, noting a significant difference between the two groups. Mean scores for BDI were 8.6 vs 14.5, for HAMD 4.0 vs 8.0, for GAF 63.3 vs 52.8 and for the World Health Organization Quality of Life Questionnaire (WHO-QoL) 55.0 vs 48.9. For neither of these scores was there a significant difference between the two groups. Davidson et al. [70] noticed positive changes in depression and anxiety by 28% and 51% respectively, except for one patient who remained severely depressed and did not benefit from DBS, requiring hospitalization for detoxification [70]. Leong et al. [68] reported a decrease in depressive symptoms by 63.5% but no effect on anxiety [68]. Most studies reported improvements in their patients’ accompanying disorders when abstinence or reduced consumption were achieved.

With regards to QoL, most publications did not report any or enough detail (*n* = 22) [47–60, 62–64, 66, 68–71], at times providing brief subjective mentions of the patients’ status. Four studies made use of QoL assessment tools, including the Modular System for Quality of Life [61], Medical Outcomes Study 36-Item Short-Form Health Survey [65, 67], Work and Social Assessment Scale [67], or the WHO-QoL [46].

## Discussion

To our knowledge, this is the most comprehensive systematic review of the application of DBS for SUDs. Many SUD patients have received assistance via contemporary pharmaco-psychiatric interventions but success rates for reduction in substance craving, seeking and consumption have not been satisfactory. Socioeconomic costs are significant and increase exponentially over time as interrelated complications present a cumulatively detrimental effect. As individual lives are devastated, becoming refractory to treatment, the whole community pays the price for inaction in health care expenses, criminality and justice system costs including law enforcement, motor vehicle accidents and lost productivity [6, 12, 13, 15, 17]. A new approach is urgently required, to reduce relapse rates and improve the QoL of severely addicted individuals.

### DBS as a therapeutic alternative

Most publications reported reduction in cravings, which is a fundamental initial step in countering substance dependence. Only 26.8% of the patients achieved abstinence but 49.3% demonstrated significant reduction in consumption, with some from this subset also showing sustained periods of abstinence. It should be noted that outcomes were reported within the context of the time frame set for the respective studies, ranging from 10 months to 8 years. Ideally, patients are followed-up for periods of time that extend to at least 5 years to determine whether abstinence has been achieved, and even then, the risk of relapse cannot be considered completely averted [72, 73]. Only two studies included in this review followed patients beyond 5 years [56, 62]. The patient in Zhou et al. [56] was followed for 6 years and was abstinent all throughout. From the five patients in Muller et al. [62] only one patient was abstinent after 8 years.

Some patients did not extinguish addictions and did not show a reduction in consumption despite admitting to reduced cravings [49, 52, 60, 62–65, 68, 70, 71]. In some instances, these cases were explained by the researchers as originating from displaced leads [52, 60, 62] and in others it was remarked how the patients had not overcome comorbid neuropsychiatric pathologies that compromised recovery [64, 70], or were unmotivated to overcome their dependence [49, 71]. Regardless, these ultimately represent a failure of the intended therapy to provide the expected benefit. The RCT [46] is expected to deliver the highest level of evidence on the efficacy of DBS in treating SUDs. However, it only recruited 12 patients despite having been planned for 30. This is attributable to the application of strict recruitment criteria, with possible influence of socioeconomic factors that excluded many patients refractory to conservative therapy. Ultimately, this diminishes the analytical capacity of the study and reduces generalizability to the larger population. There was no statistically significant difference in selected outcome between the experimental and control groups.

When considering DBS as a therapeutic alternative in the treatment of SUDs it should also be remembered that DBS is a costly and invasive surgical intervention and as such it carries risks, including infection, device failure and side effects such as insomnia, hypomania and impulsivity. Serious complications include the possibility of intracerebral hemorrhage or stroke, although these are rare [74]. Moreover, implantable pulse generators require replacement, with non-rechargeable models lasting for longer [75]. Long-lasting success of the therapeutic process also necessitates joint specialist collaboration within dedicated centers. Consequently, when considering the increasing number of patients not responding to pharmaco-psychiatric treatment for SUD, the possibility of securing all the resources required to make a significant impact on this sociocultural phenomenon seems unlikely or, at best, conducive to only marginal benefits to a small percentage of patients.

### DBS targets in treating SUDs

Most studies focused on targets around the ventral capsule, including the BNST and especially the NAcc. This has been deemed the safest and most effective approach, as suggested by numerous preclinical studies [29]. Located in the basal forebrain and positioned between the caudate and the putamen, the NAcc is a substructure, and the main component, of the ventral striatum within the basal ganglia [29]. The dual characteristics of the NAcc suggest that the core is involved in the initiation of cue-primed substance attainment and the shell is concerned with reinforcement of that behavior [76, 77]. The NAcc plays a prominent role in the mesocorticolimbic reward circuitry due to its neuronal connections and the function it exerts in the formation of addiction, making its direct stimulation via DBS effective in reducing the craving for substances of abuse and preventing relapse [78, 79].

It is very likely that multiple mechanisms account for the effects of DBS, including neural oscillation, electrical stimulation of nerves and neurochemical effects on a local and a wider neural network, neuroplasticity, neurogenesis, and neuroprotection modulation [80]. Newer, improved protocols of DBS delivery are being refined, intended to enhance its therapeutic effects for multiple clinical applications, including the treatment of SUDs [81]. Different forms of stimulation, such as adaptive, burst, and coordinated reset, and a deeper understanding of neuronal circuit architecture can help create more tailored therapy, better adjusted at inducing changes in the targeted circuity and in reaching the intended therapeutic outcome [82].

### A vulnerable population

Most patients present co-occurring neuropsychiatric conditions which, to a certain extent, hinder their recovery from SUDs. OCD, depression and anxiety are among the most encountered psychiatric pathologies. For some patients, reduced cravings helped address their accompanying neuropsychiatric issues. The ensuing synergistic effect boosted recovery, resulting in improved health, weight, sexual life, mood and social life [50, 67]. In other instances, despite reporting reduced cravings, patients struggled with comorbid disorders, impeding rehabilitation. Some consumed other substances of abuse because of “boredom” or “habit”, demonstrating a tendency for poly-substance abuse [49, 61, 64, 70]. While this might hint at potential neural circuitry compromise originating from prolonged exposure to the substance of interest, it also opens a window onto the relevance of cues derived from the social context of the individual. A patient with SUD will more readily relapse in the presence of others engaging in consumption or within environments where in the past the individual would have consumed the substance. Impactful life events can be potent psychosocial stressors that also facilitate relapse. The way a patient conceives their relationship with the substance of abuse and whether there is intrinsic acceptance that an addictive pattern of behavior exists, is also key to successful treatment.

Patients should be placed in context, considering the multidimensionality of their reality. Environmental cues, comorbid pathologies, their thought processes and their socioeconomic situation should be considered. With a vulnerable patient population such as the one amenable for DBS intervention the risk of the individual being alienated from social support structures and potential financial ruin runs higher as the patient gets entangled in physiological reward circuitry processes over which control only grows more ephemeral. Therapeutic targets should include prompt reinsertion into a social group, re-learning an approach to creating and strengthening social relationships, and the acquisition of skills to assist with reintroduction into a workplace environment [83].

### Study limitations

The main limitation of the study is the small sample size of the patient population across the publications retrieved, with most being case studies reporting on one single patient. Recent trials engaged more participants but even the first RCT [46] presents limited statistical power, with just 12 subjects involved. Publication bias needs to be considered as small sample sizes, and the absence of randomization and a control group make research more vulnerable for this [84, 85]. Studies not available in English were not considered for review. Inclusion criteria could not be severely restrictive to capture as much of the limited data available on the subject as possible. Outcomes for the primary intent of the research varied across the studies, making comparisons imprecise. Measurements were often based on self-reports and the methodologies employed often differed between studies. Blinding and randomization were only utilized in three studies. Parallel variables that could influence the outcome of research into alleviating dependence, such as concomitant neuropsychological pathologies and relevant social and emotional cues, were not always reported and measured.

## Conclusion

Clinical studies suggest that DBS as a therapeutic option for treatment-refractory SUDs has not yielded high enough remission rates that would currently justify substituting more readily available, cheaper, and less invasive pharmaco-psychiatric approaches. Studies have reported satisfactory efficacy levels in reducing cravings and heavy consumption. This is an improvement on current treatment modalities, which assist with detoxification but have often proven limited in preventing relapse. However, achieving and maintaining abstinence via DBS has also proven difficult, indicating learned behavior and the engagement of areas of the brain that are untapped or insufficiently modified with present day DBS techniques. Use of DBS as mainstream therapy for SUDs faces several challenges given the considerable resources required, and this contrasts with the level at which SUD patients are increasing in number worldwide. More research is necessary, with heightened methodological rigor and standardization of parameters, including patient selection, objective outcome assessments, longer follow-up periods and a pre-established surgical protocol. The development of enhanced techniques and more specific procedure methodologies would be expected to improve therapeutic delivery of DBS for SUDs, overcoming the current limitations. SUDs are difficult to address for physicians, the patients, and their significant others. They hold hostage invaluable human potential which would otherwise be employed productively for the benefit of society. Use of DBS in this domain holds promise, perhaps at present primarily as a vehicle for gaining better understanding of addiction dynamics in the brain, investigating the neural circuits and the biological mechanisms concerned, with the prospect of applying new protocols that will augment the efficacy of DBS in treating SUDs. This study is intended to encourage further research into the potential of DBS to address addiction disorders and inform future therapy.
